# Myeloid-derived suppressor cells in peripheral blood as predictive biomarkers in patients with solid tumors undergoing immune checkpoint therapy: systematic review and meta-analysis

**DOI:** 10.3389/fimmu.2024.1403771

**Published:** 2024-05-24

**Authors:** Maximilian Möller, Vanessa Orth, Viktor Umansky, Svetlana Hetjens, Volker Braun, Christoph Reißfelder, Julia Hardt, Steffen Seyfried

**Affiliations:** ^1^ Department of Surgery, Medical Faculty Mannheim, University Medical Center Mannheim, Heidelberg University, Mannheim, Germany; ^2^ Department of Dermatology, Venereology and Allergology, University Medical Center Mannheim, Heidelberg University, Mannheim, Germany; ^3^ Skin Cancer Unit, German Cancer Research Center (DKFZ), Heidelberg, Germany; ^4^ German Cancer Research Center (DKFZ)-Hector Cancer Institute, University Medical Centre Mannheim, Mannheim, Germany; ^5^ Department of Biometry and Statistics, Medical Faculty Mannheim, University Medical Center Mannheim, Heidelberg University, Mannheim, Germany; ^6^ Department of Library and Information Sciences, Medical Faculty Mannheim, Heidelberg University, Mannheim, Germany

**Keywords:** immune checkpoint inhibitors, immunotherapy, myeloid-derived suppressor cells, MDSC, neoplasms, solid malignancies, prognosis, biomarkers

## Abstract

**Background:**

Immunotherapeutic approaches, including immune checkpoint inhibitor (ICI) therapy, are increasingly recognized for their potential. Despite notable successes, patient responses to these treatments vary significantly. The absence of reliable predictive and prognostic biomarkers hampers the ability to foresee outcomes. This meta-analysis aims to evaluate the predictive significance of circulating myeloid-derived suppressor cells (MDSC) in patients with solid tumors undergoing ICI therapy, focusing on progression-free survival (PFS) and overall survival (OS).

**Methods:**

A comprehensive literature search was performed across PubMed and EMBASE from January 2007 to November 2023, utilizing keywords related to MDSC and ICI. We extracted hazard ratios (HRs) and 95% confidence intervals (CIs) directly from the publications or calculated them based on the reported data. A hazard ratio greater than 1 indicated a beneficial effect of low MDSC levels. We assessed heterogeneity and effect size through subgroup analyses.

**Results:**

Our search yielded 4,023 articles, of which 17 studies involving 1,035 patients were included. The analysis revealed that patients with lower levels of circulating MDSC experienced significantly improved OS (HR=2.13 [95% CI 1.51–2.99]) and PFS (HR=1.87 [95% CI 1.29–2.72]) in response to ICI therapy. Notably, heterogeneity across these outcomes was primarily attributed to differences in polymorphonuclear MDSC (PMN-MDSC) subpopulations and varying cutoff methodologies used in the studies. The monocytic MDSC (M-MDSC) subpopulation emerged as a consistent and significant prognostic marker across various subgroup analyses, including ethnicity, tumor type, ICI target, sample size, and cutoff methodology.

**Conclusions:**

Our findings suggest that standardized assessment of MDSC, particularly M-MDSC, should be integral to ICI therapy strategies. These cells hold the promise of identifying patients at risk of poor response to ICI therapy, enabling tailored treatment approaches. Further research focusing on the standardization of markers and validation of cutoff methods is crucial for integrating MDSC into clinical practice.

**Systematic Review Registration:**

https://www.crd.york.ac.uk/prospero/display_record.php?ID=CRD42023420095, identifier CRD42023420095.

## 1 Introduction

Despite modern therapies, cancer is still one of the most common causes of death in industrialized countries. For example, in 2019, solid tumors such as tracheobronchial lung cancer, prostate and colon cancer were among the leading causes of death worldwide from cancer in men while it was breast, colon cancer and tracheobronchial lungs in women (1). The approval of immune checkpoint inhibitor (ICI) treatments, by the Food and Drug Administration in 2011provides alternative therapies to the standard chemotherapy regimens, particularly for the treatment of solid tumor malignancies It has been shown that T cells become anergic in cancer patients due to the interaction of programmed death -1 (PD-1) or cytotoxic-T-lymphocyte-associated protein-4 (CTLA-4) upregulated on activated T cells with their ligands PD-L1 and CD80 or CD86 respectively. Blocking this interaction could result in regaining anti-tumor T cell functions (2, 3).

With the introduction of Ipilimumab in the treatment of malignant melanoma, the median overall survival was increased from 6.4 months to 10 months compared to the control group (4). The survival curve in a cohort of patients with non-resectable malignant melanoma treated with ipilimumab reached a plateau between 20–26% after three years, indicating a long-term response (5). Another breakthrough was found in the treatment of non-small-cell lung carcinoma. Here, recent studies have shown that immunotherapy combined with chemotherapy already shows a survival advantage in the first line therapy compared to the single chemotherapy (median overall survival after 12 months: 69.2% in the combination group and 49.4% in the chemotherapy group) (6).

Despite these advancements, a subset of patients either fails to respond initially or loses responsiveness over time to such therapies. The search for explanations has increasingly focused on immunosuppressive mechanisms, including the role of myeloid-derived suppressor cells (MDSC). Studies have indicated an inverse relationship between the prognosis of solid tumor patients and the presence of immunosuppressive cell types such as MDSC and regulatory T-cells (Treg) within the tumor microenvironment (TME) and peripheral blood (7–9).

MDSCs represent a heterogeneous population of myeloid cells known for their immunosuppressive activities. They originate from immature myeloid cells that fail to differentiate under chronic inflammatory conditions, such as cancer (10, 11). In addition, normal mature myeloid cells could be converted into MDSC in cancer patients (12, 13). MDSCs are categorized into two subpopulations based on their phenotypic characteristics: monocytic MDSCs (M-MDSCs) and polymorphonuclear MDSCs (PMN-MDSCs). M-MDSCs are identified by the expression of surface markers CD11b^+^CD14^+^HLA-DR^low/-^CD15^-^, with CD33 also serving as an alternative marker to CD11b. This subgroup is morphologically comparable to monocytes. On the other hand, PMN-MDSCs, which express CD11b^+^CD14^-^CD15^+^(CD66 as an alternative to CD15) markers, are morphologically akin to neutrophils (10, 11).

The discovery of Lectin-type oxidized LDL receptor 1 (LOX-1) as a specific ligand has refined the identification and separation of these cell types, facilitating a more accurate characterization and understanding of their roles within the tumor microenvironment (TME) and systemic circulation (14). A standardized gating strategy to identify M-MDSCs, based on common morphological criteria such as CD14^+^ and HLA-DR expression, has been established recently, noting that functional examination of the immunosuppressive properties of MDSCs is the safest way to identify them (10). This advancement in methodology has been crucial for consistent and reproducible analysis of MDSC populations across various studies.

The suppressive mechanisms of MDSC include inhibiting T cells and other components of immune systems to facilitate tumor growth and survival (11). One of the major mechanisms of MDSC-mediated immunosuppression is linked to the upregulation of programmed cell death ligand 1 (PD-L1) interacting with its receptor PD-1 expressed on tumor-infiltrating T cells (10, 15),

They are also capable to inhibit anti-tumor T cell functions via production of nitric oxide (NO) and reactive oxygen species (ROS) as well as by upregulation of arginase 1 and Indolamin-2,3-Dioxygenase (11, 16–19).

They also interact synergistically with regulatory T cells (Tregs), promoting their expansion within the TME via the CD40 receptor (20). This interaction highlights the complex network of immunosuppressive pathways that contribute to tumor growth and survival.

Given these extensive immunosuppressive capabilities, MDSC subpopulations represent potential biomarkers for predicting patient outcomes, including responses to immunotherapies (11, 21–23). Furthermore, the recruitment of MDSCs from the bone marrow to the TME, driven by various cytokines (24) suggests that an early increase in circulating MDSC levels could serve as a negative prognostic indicator.

Despite existing research demonstrating a correlation between high levels of MDSCs in peripheral blood and adverse outcomes in solid tumors (9), the specific impact of MDSCs on the efficacy of immune checkpoint inhibitor (ICI) therapy remains underexplored. To date, no systematic review or meta-analysis has separately assessed the predictive value of circulating MDSCs on the response to ICI therapy in patients with solid tumors. This gap in the literature underscores the need for a comprehensive analysis that can elucidate the influence of circulating MDSC populations on overall survival and therapeutic response, thereby informing clinical decision-making and potentially guiding the development of more effective treatment strategies. The goal of this meta-analysis is to address this need by examining the relationship between MDSC levels in peripheral blood and patient outcomes in the context of ICI therapy.

## 2 Methods

This study was conceived as a meta-analysis to investigate whether elevated levels MDSCs and their subpopulations in peripheral blood serve as predictive markers for the response to immune checkpoint inhibitor (ICI) therapy or survival outcomes in patients with solid tumors. The PICO scheme for our research question was defined as:

- Population. Patients with solid tumors treated with ICI
- Intervention: measurement of MDSC in patients’ peripheral blood by flow cytometry
- Comparison: high concentrations of MDSC compared to low concentrations of MDSC
- Outcome: Progression-free survival and overall survival

The study protocol was prospectively registered with PROSPERO (registration number CRD42023420095), adhering to the Preferred Reporting Items for Systematic Reviews and Meta-Analysis Protocols 2020 (PRISMA-P) guidelines, which underpin both the protocol and the manuscript structure (see **Supplementary Table 2** for details).

### 2.1 Search strategy

The literature search was conducted in the PubMed and EMBASE databases from January 2007 to November 2023, utilizing the PubMed and EMBASE databases. A detailed search strategy was developed in collaboration with a medical librarian, incorporating terms and synonyms related to immune checkpoint inhibitors and “myeloid-derived suppressor cells” [MeSH], utilizing both OR and AND Boolean operators for term combination. Additionally, Google Scholar was employed to identify grey literature, and the reference lists of relevant articles were reviewed to uncover further studies.

### 2.2 Inclusion criteria

Inclusion criteria mandated that studies: (1) were prospective or retrospective cohort studies, clinical trials, or randomized controlled trials; (2) included patients diagnosed with solid neoplasms; (3) involved treatment with an immune checkpoint inhibitor; (4) measured MDSC levels in peripheral blood at a minimum of two time points, one of which must be prior to therapy initiation; (5) used cutoff values for MDSC levels to stratify patients; and (6) performed a correlation analysis with survival or other outcome parameters, including either (7) hazard ratios with 95% confidence intervals or provided sufficient data for their calculation.

### 2.3 Exclusion criteria

Excluded were studies that: (1) were reviews, case reports, animal studies, or *in vitro* studies; (2) did not measure MDSC levels using flow cytometry or measured them peritumorally or directly within tumor tissues; (3) targeted MDSCs directly as a therapeutic intervention; (4) provided insufficient data for hazard ratio calculations.

The eligible studies were screened in full text by two authors (MM, VO) with discrepancies resolved via a third author (SS).

### 2.4 Data extraction

The data were collected by both authors independently in a data matrix that included the first author, year of publication, country of origin, number of patients and age (median and/or range) of the study population, tumor type and stage, type of therapy (immune checkpoint inhibitor with target) MDSC subpopulation, MDSC markers, cutoff values, and method of cutoff value determination, observed endpoints. We extracted hazard ratios with 95% confidence intervals for the endpoints overall survival (OS), progression free survival (PFS), disease free survival (DFS). If these were not specified, we calculated the hazard ratios according to method of Tierney (25) by estimating the necessary data from the Kaplan Meier curves. Alternatively, we extracted the HR from other sources if the data were already calculated there.

### 2.5 Risk of bias assessment

Using the QUIPS tool, one author (MM) assessed the risk of bias of the included studies. A second author (VO) independently reviewed the assessment. Disagreements were resolved by a third author (SS). The tool contains six categories of bias due to study participation, study attrition, prognostic factor measurement, outcome measurement, adjustment for other prognostic factors and bias due to statistical analysis and reporting. In each category, the authors could choose between low, moderate and high risk of bias (26).

### 2.6 Statistical analysis

For the statistical analysis, we used RevMan 5.4 (Review Manager Version 5.4. The Cochrane Collaboration, 2020) and Comprehensive Meta-Analysis software Version 4 (Biostat, Englewood, NJ 2022). We weighted them according to the generic inverse variance method. A hazard ratio >1 defined a preference for a low MDSC level at baseline. To detect heterogeneity, we used a χ² test and the I² value, which were considered significant if the χ² test assumed a value of P<0.1 or I²>50% (27). Subgroup and sensitivity analyses further explored heterogeneity, while publication bias was assessed visually with a funnel plot and quantitatively via the Egger test. The Duval and Tweedie’s trim-and-fill method was applied in cases of detected asymmetry, with a significance threshold set at P<0.05 (28). If the heterogeneity was significant, we used the random-effects model; otherwise, we used the fixed-effects model.

## 3 Results

### 3.1 Study characteristics

**Figure 1:**
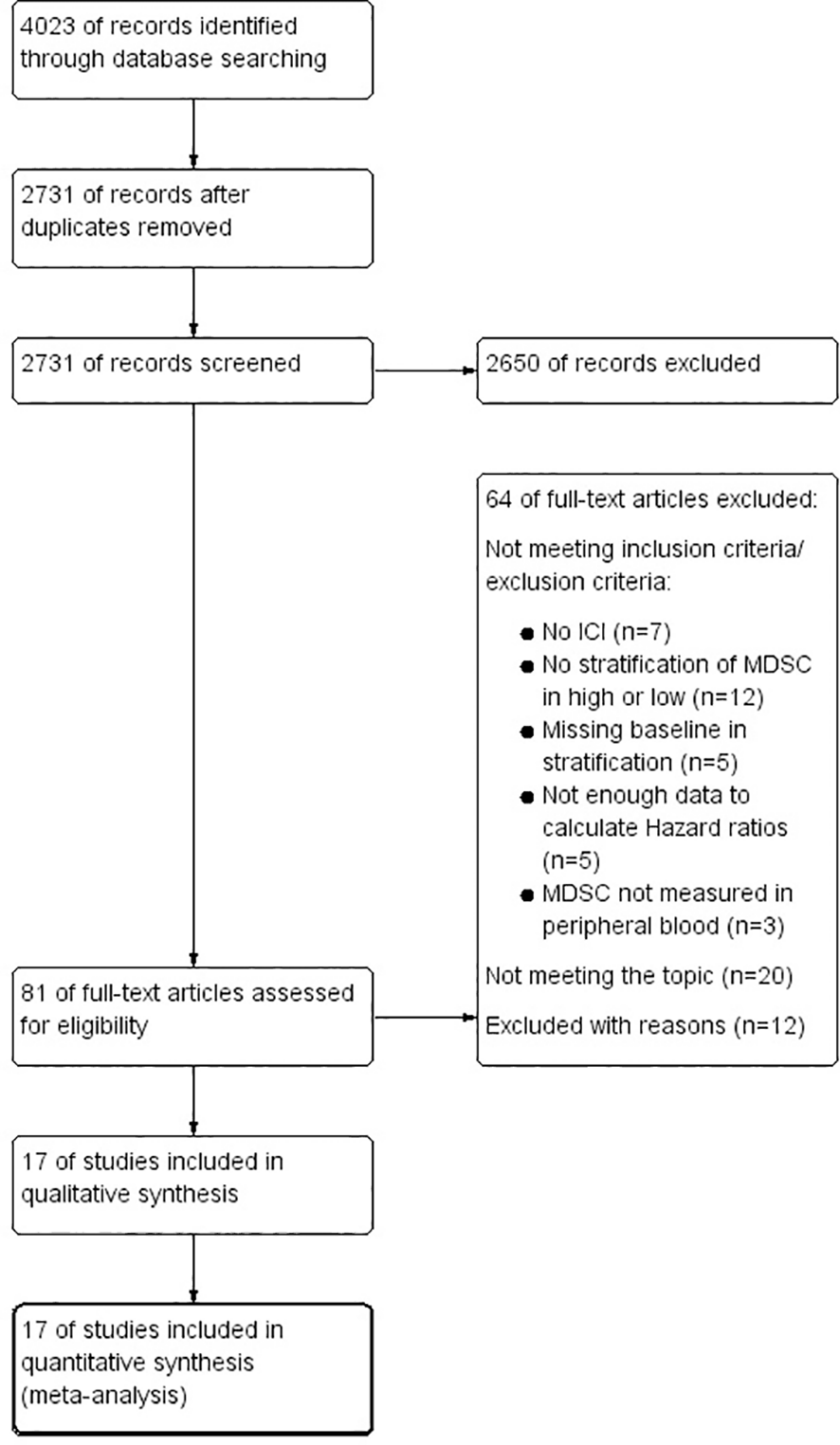
PRISMA Flow Chart, illustrating the study selection process.

In our comprehensive search across three databases (PubMed, EMBASE, Google Scholar), we initially identified 4,023 articles. Upon removal of 1,197 duplicates, 2,731 articles remained for consideration. The initial screening of titles and abstracts facilitated the exclusion of 2,650 articles deemed not relevant to our research objectives. Further detailed examination of the full texts led to the exclusion of additional articles for various reasons: 12 articles were excluded due to lack of stratification of MDSC levels into high or low categories; 5 articles were omitted because they failed to collect baseline data; 7 articles were excluded for not incorporating immune checkpoint inhibitor (ICI) therapy; 5 articles were disregarded due to insufficient data for calculating Hazard ratios; 3 articles were eliminated because they did not measure MDSC in peripheral blood; and 32 articles were excluded for other reasons or because they did not align with the study’s focus. Ultimately, 17 studies (29–45) were selected for inclusion in our meta-analysis, as illustrated in the PRISMA flow diagram (**Figure 1**).

These 17 studies collectively encompassed 1,035 patients, with the majority being melanoma cases (10 studies involving 720 patients). The next most significant group was patients with non-small cell lung cancer (NSCLC), represented by 207 patients across 3 studies. Other cancer types included in the analysis were prostate cancer (2 studies with 45 patients) and urothelial carcinoma (1 study with 30 patients), along with a study that pooled data from patients with various solid tumors (33 patients across 11 entities). Regarding ICI specificity, 8 studies targeted PD-1/PD-L1 exclusively, involving 428 patients; 5 studies focused solely on CTLA-4, including 359 patients; and 4 studies, comprising 248 patients, pooled effect measurements for patients treated with either PD-1/PD-L1 or CTLA-4 antibodies.

A more detailed breakdown of these studies is provided in **Table 1**. Out of the included studies, 15 reported data on OS, and 9 included PFS data. The analysis of MDSC subtypes revealed that 2 studies examined total MDSC levels, 2 focused exclusively on PMN-MDSC, and 7 investigated monocytic MDSC (M-MDSC). Additionally, 6 studies presented data relevant to both PMN-MDSC and M-MDSC subgroups, offering a comprehensive overview of the impact of these immune cells on patient outcomes in the context of ICI therapy.

**Table 1:** Main characteristics of the 17 studies qualified to be included in this meta-analysis.

Author, year	Country	Cancer type	Stage	Immunotherapeutic agent (1=PD-1, 2=CTLA4, 3 PDL-1)	Sample size	Age_a_	MDSC type	MDSC marker	Cutoff method	Cutoff value	Outcome	Data availability
Gaißler 2023 (31);	Germany	Melanoma	IV	(1)Pembrolizumab, (1) Nivolumab single or + (2) Ipilimumab	141	64	M-MDSC	M-MDSC: Lin- CD11b+/CD14+/CD33+/HLA Drlow	other ‡	18,1% (%total mononuclear leucocytes)	PFS, OS	extracted paper
Tomela, 2023 (29);	Poland	Melanoma	III-IV	(1)Nivolumab, (1)Pembrolizumab	46	63 (32–92)	total MDSC M-MDSC PMN-MDSC	MDSC: CD11b+/HLA-DR−/low/CD33+, M-MDSC: CD14+/CD33high/CD11b+/HLA-DR−/low and PMN-MDSC: CD66b+/CD33dim/CD11b+/HLA-DR−/low	Median	7.1%(total), 3% (PMN-MDSC), 4.1% (M-MDSC) (%alive PBMC)	PFS	calculated
Petrova, 2023 (30);	Germany	Melanoma	III-IV	(1)Pembrolizumab, (1)Nivolumab single or + (3)Ipilimumab	29	64(41–84),	M-MDSC, PMN-MDSC	M-MDSC: HLA-DRlow/−CD33highCD14+ PMN-MDSC: HLA-DRlow/−CD33dimCD66b+Lin−;	Median	0.54% (PMN-MDSC) 0.73% (M-MDSC) (%alive PBMC)	PFS, OS	Extracted paper♦
Girardi, 2022 (32);	USA	Urothelian carcinoma	IV	(1)Nivolumab+Cabozantinib	30	64.5 (47–80)	M-MDSC PMN-MDSC	M-MDSC: CD14+ CD11b+ HLA–DRlow/– CD15–. PMN-MDSC: CD14− CD11b+ CD15+;	Median	NR	PFS, OS	calculated
Bronte, 2022 (33);	Italy	NSCLC	III-IV	(1)Pembrolizumab, (1)Nivolumab (3)Atezolizumab, Combination	22	70.1 (64.8–75.0)	M-MDSC	M-MDSC: CD14 + HLA-DR − /lowCD11b + CD33 +	Median	1.9% (%CD45+)	PFS, OS	extracted paper
Araujo, 2021 (35);	Denmark	Solid tumor	mixed	mixed (1,2,3)	33	60 (36 -75)	M-MDSC	M-MDSC: CD14+ CD3- CD19- HLA-DR low, CD56-	Median	NR (% alive PBMC)	PFS, OS	extracted paper
Krebs, 2021 (34);	Germany	Melanoma	III-IV	(2)Ipilimumab (1,2)Ipilimumab/Nivolumab (1)Pembrolizumab, (1)Nivolumab	45	70 (27–86)	PMN-MDSC	PMN-MDSC: CD15+CD33+;	Median	0.5% (% alive PBMC)	OS	Calculated
de Coaña, 2020 (37);	Sweden	Melanoma	IV	(1)Nivolumab, (1)Pembrolizumab	36	68.5 (37–83)	M-MDSC PMN-MDSC	M-MDSC: CD14+HLA-DRlow/-; PMN-MDSC: NR	cutoff finder software	0.09% (PMN-MDSC), 10.70% (M-MDSC), (% alive PBMC)	OS,	extracted paper
Koh, 2020 (39);	Korea	NSCLC	I-IV	(1)Nivolumab, (1)Pembrolizumab	132	62 (34–88)	M-MDSC PMN-MDSC	PMN-MDSC: Lin− CD15+ CD14− CD11b+ HLA-DR−/low; M-MDSC: Lin− CD15− CD14+ HLA-DR−/low	Median	NR	PFS, OS	extracted paper
Passaro et al., 2020 (38);	Italy	NSCLC	III-IV	(1)Nivolumab	53	64 (56–70)	PMN-MDSC	PMN-MDSC: SSClow Lin−/HLA-DR−/lowCD33+/CD13+/CD11b+/CD15+/CD14−	other	6 cell/μL	PFS, OS	extracted paper
Karzai, 2018 (40),	USA	Prostate Cancer	IV	(1)durvalumab + olaparib	17	66 (45–79)	MDSC	MDSC: CD3+−CD19−CD56−HLA-DR− CD11b+CD33+	Median	1.26% (% total viable cells)	PFS	calculated
de Coaña, 2017 (36);	Sweden	Melanoma	IV	(2)Ipilimumab	43	(23–80)	M-MDSC, PMN-MDSC	PMN-MDSC: Lin-CD14- CD11b+ CD33+ CD15+ HLA-DRlo/neg; M-MDSC: CD14+ HLA-DRlo/neg	cutoff finder software	2.3%(PMN-MDSC); 18.6% (M-MDSC), (% alive PBMC)	OS	extracted paper
Sade-Feldman, 2016 (42);	Israel	Melanoma	IV	(2)Ipilimumab	56	60.7	MDSC	MDSC: CD33+CD11b+HLA-DR-	Data distribution	55.5% (as the CD33+CD11b+ (%) of gated HLA-DR− cells)	OS	calculated
Weber, 2016 (41);	USA	Melanoma	III-IV	(1)Nivolumab	92	60	M-MDSC	M-MDSC: Lin-CD11b+/CD14+/HLA Drlow	Median	12.6% (%alive PBMC)	OS	calculated
Martens, 2016 (43);	Europe (multicentral)	Melanoma	IV	(2)Ipilimumab	209 (MDSC measured n=164)	58	M-MDSC	MDSC: Lin−CD14+HLA-DR+−/niedrig	Other	5.1% (NR)	OS	extracted paper
Santegoets, 2014 (44);	Netherlands	Prostate Cancer	IV	(2) Ipilimumab + GVAX	28	NR	M-MDSC	M-MDSC: Lin- CD14+ HLA-DR-/low,	Cox regression	0.3% (NR)	OS	extracted paper
Kitano, 2014 (45);	USA	Melanoma	III-IV	(2)Ipilimumab	68	62 (34–83)	M–MDSC	M-MDSC: Lin- CD14+CD11b+ HLA-DRlow/-	Log-Rank-Statistic	14.9% (%HLA-DR low/− in Lin-CD14+CD11b+)	OS	extracted paper

United states of America (USA); non-small cell lung cancer (NSCLC); GVAX vaccine; myeloid-derived suppressor cells (MDSC); monocytic MDSC (M-MDSC); polymorphonuclear (PMN-MDSC); Peripheral blood mononuclear cells (PBMC); Programmed Cell Death Protein 1 (PD-1), Programmed cell death ligand-1 (PD-L1), cytotoxic T-lymphocyte-associated Protein 4 (CTLA-4), not reported (NR).

### 3.2 Progression-free survival

**Figure 2:**
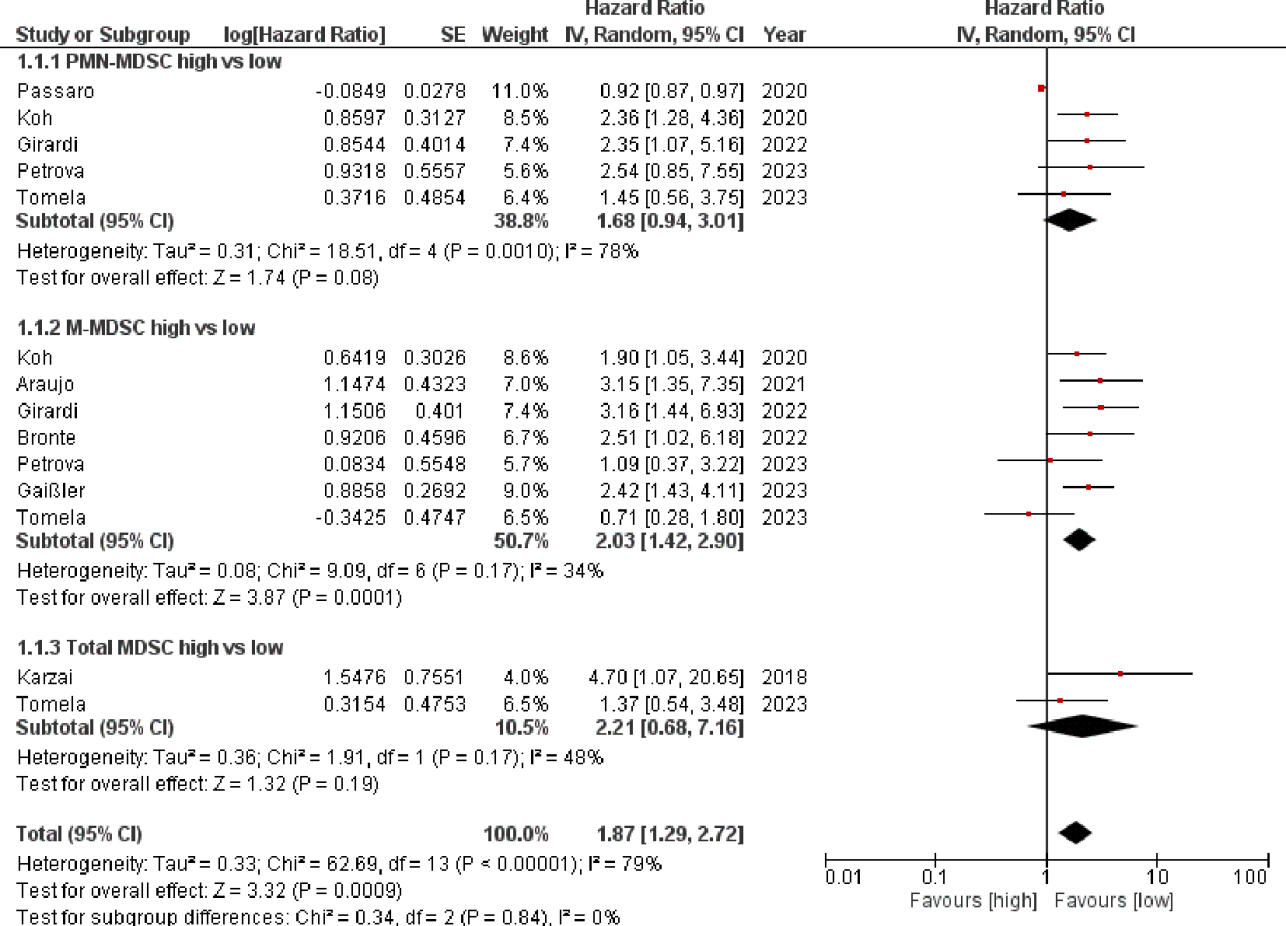
Forest plot illustrating the impact of myeloid-derived suppressor cell levels on progression-free survival in patients receiving immune checkpoint inhibitor therapy.

A high baseline MDSC value indicated a poorer response to ICI, according to PFS. The HR was 1.87 [95%CI 1.29–2.72] with an I^2^ of 79%. Here, however, only the M-MDSC achieved a significant result (HR= 2.03 [95% CI 1.42–2.90]) with a moderate heterogeneity I^2 =^ 34% %. The other three populations were not significant (PMN MDSC HR=1.68 [95% CI 0.94–3.01] I^2 =^ 78%); total MDSC HR=2.21 (95%CI [0.68–7.16] I^2 =^ 48%). Due to the overall high heterogeneity, the HRs were also calculated here using a random effects model (**Figure 2**).

### 3.3 Overall survival

**Figure 3:**
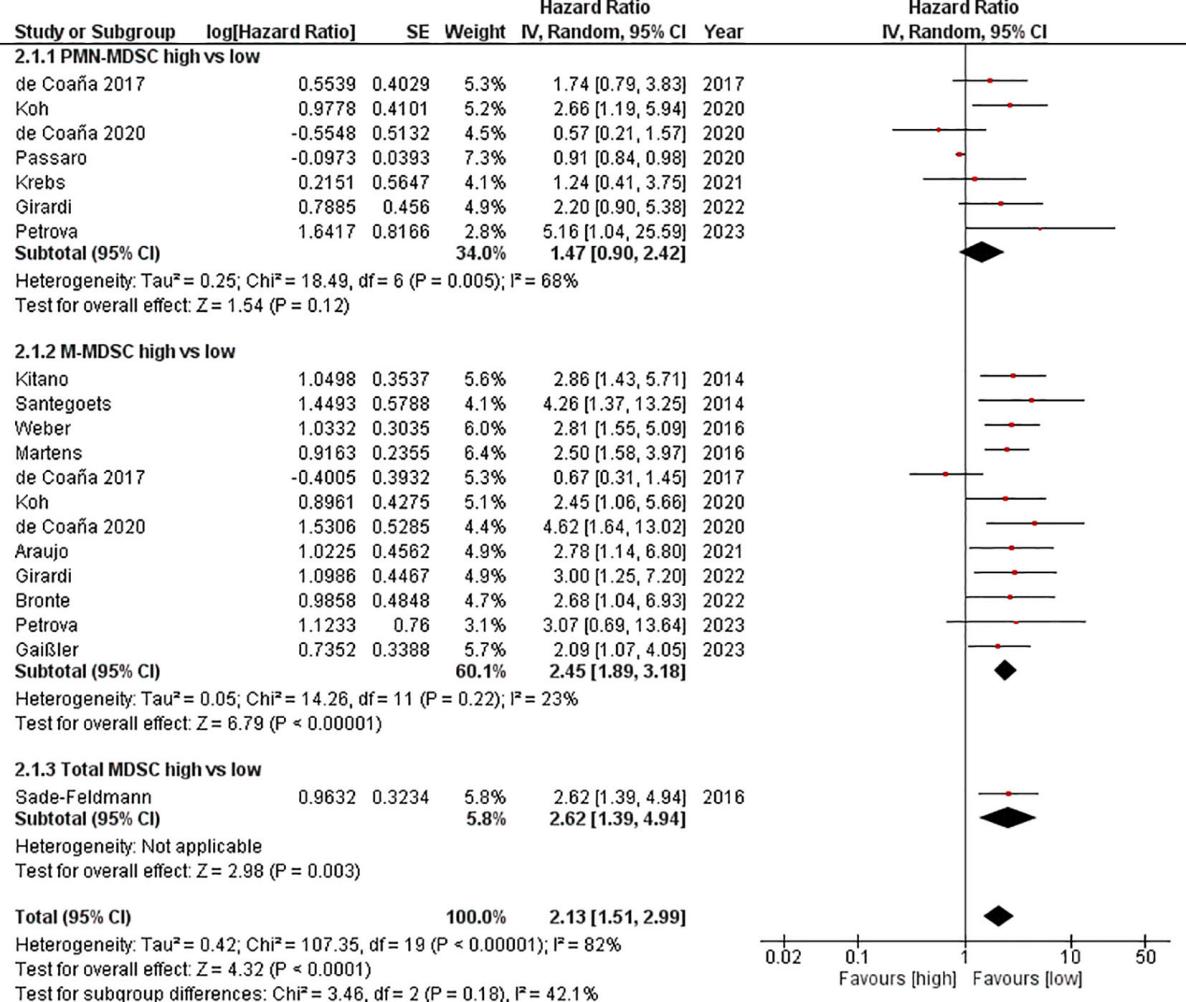
Forest plot illustrating the impact of myeloid-derived suppressor cell levels on overall survival in patients receiving immune checkpoint inhibitor therapy.

OS was lower with a high MDSC baseline value. This resulted in an HR=2.13 [95% CI 1.51–2.99] for OS after pooling the studies. The heterogeneity amounted to I^2^ = 82%. However, only the M-MDSC reached a significant level. We calculated an HR=2.45 [95% CI 1.89–3.18] I^2^ = 23%) for the M-MDSC. For the PMN-MDSC population, the HR was 1.47 [95%CI 0.90–2.42]. This resulted in I^2^ = 68%. Overall, the heterogeneity was relatively high, which is why the HR was calculated using the random effects model (**Figure 3**).

### 3.4 Subgroup analysis

To delve deeper into the heterogeneity observed within our meta-analysis, we conducted detailed subgroup analyses focusing on variables such as geographic region, patient cohort size, type of cancer, stage of cancer, method and value used for cutoff determination, and the specific immune checkpoint target (PD-1/PD-L1 versus CTLA-4). Stratification for the number of patients and cutoff values was based on the median values within each respective group. The comprehensive findings of these subgroup analyses are presented in **Supplementary Table 1**. A reduction in heterogeneity was observed when studies utilized consistent methods for determining cutoff values, highlighting this as a significant factor in our analysis. The type of cancer entity did not impact heterogeneity. Predominantly, PMN-MDSC emerged as a primary source of heterogeneity, both in our primary and subgroup analyses.

In instances where a high degree of pooled heterogeneity was noted, it was often accompanied by significant heterogeneity within the PMN-MDSC subgroup, as detailed in the **Supplementary Material**. A visual inspection of the Forest plot readily identified the study by Passaro et al. as a potential primary contributor to this observed heterogeneity. Further analysis confirmed that the inclusion of Passaro et al. markedly influenced the heterogeneity levels: for the PMN-MDSC subgroup analyzing PFS, heterogeneity dramatically decreased from I²=78% to 0% upon excluding this study, resulting in an adjusted HR of 2.18 (95% confidence interval [CI]: 1.46–3.26) and a revised pooled HR of 2.10 (95% CI: 1.68–2.62) with an I² of 0%. A similar pattern emerged within the overall survival subgroup; the exclusion of Passaro et al. halved the heterogeneity from I²=68% to 39%, with an HR of 1.73 (95% CI: 1.04–2.88), leading to a pooled HR of 2.26 (95% CI: 1.81–2.83) and an I² of 27%.

This significant reduction in heterogeneity, particularly in the PFS PMN-MDSC population, from I²=78% to a null value (I²=0%) following the removal of Passaro et al., underscores the substantial impact this study had on the heterogeneity levels. Similarly, in the OS analysis, the heterogeneity within the PMN-MDSC subgroup was notably reduced by half (from I²=68% to 39%), with the pooled HR adjusting to 2.26 (95% CI: 1.81–2.83) and an I² of 27%. These findings highlight the critical influence of specific studies on the heterogeneity of meta-analytic outcomes and underscore the importance of scrutinizing individual study contributions to the OS.

In addition, we explored the role of MDSCs as prognostic markers across various subgroups:

As shown in **Table 2**, MDSCs consistently demonstrated robust prognostic value for OS across all subgroups, except for non-small cell lung cancer (NSCLC), where the HR was 1.85 [95% CI 0.88–3.90]. For PFS, MDSCs showed strong predictive value, particularly in patients with advanced (stage IV) cancer, with an HR of 2.66 [95% CI 1.84–3.86]. Notably, MDSCs maintained low heterogeneity and high predictive value when the median cutoff method was employed.

**Table 2:** Subgroup analysis of myeloid-derived suppressor cell and progression free survival or overall survival.

Subgroup	No Studies	No Patients	Random/ Fixed Model	PFS: pooled HR [95%CI]	I²=	P Value	Random/ Fixed Model	OS: pooled HR [95%CI]	I²=	P-Value
Melanoma	10	720	Fixed	1.66 [1.18, 2.33]	24%	0.003	Random	2.03 [1.46, 2.83]	51%	< 0.001
NSCLC	3	207	Random	1.68 [0.90, 3.13]	84%	0.10	Random	1.85 [0.88, 3.90]	82%	0.11
Cancer stage IV	8	515	Fixed	2.66 [1.84, 3.86]	0%	<0.001	Random	2.01 [1.39, 2.92]	56%	< 0.001
PD-1/PDL-1	8	428	Random	1.75 [1.15, 2.67]	77%	0.009	Random	2.04 [1.20, 3.48]	84%	0.009
CTLA-4	5	359		N/A	N/A	N/A	Random	2.09 [1.34, 3.27]	58%	0.001
Study population >median (n=45)	9	797	Random	1.48 [0.95, 2.30]	79%	0.09	Random	2.08 [1.28, 3.39]	88%	0.003
Study population <Median (n=44)	8	283	Fixed	2.56 [1.80, 3.65]	0%	<0.001	Random	2.14 [1.38, 3.32]	53%	< 0.001
Cutoff method: Median	9	446	Fixed	2.04 [1.60, 2.60]	5%	<0.001	Fixed	2.60 [1.96, 3.46]	0%	< 0.001
Cutoff method: other •	8	589	Random	1.44 [0.56, 3.71]	92%	0.45	Random	1.79 [1.13, 2.85]	86%	0.01
Western countries	16	903	Random	1.83 [1.21, 2.77]	78%	0.004	Random	2.08 [1.45, 2.99]	83%	< 0.001

Overall survival (OS); progression free survival (PFS); Hazard ratio (HR); 95% Confidence interval (95% CI); [lower limit of 95% CI, upper limit of 95% CI];•including Data distribution, Log-Rank statistic, Cox regression; not applicable (N/A).

In our detailed analysis (see **Supplementary Table 1** for details), M-MDSC were identified as robust predictive markers for both OS and PFS in nearly all examined subgroups. In NSCLC specifically, M-MDSCs were predictive and prognostic for both outcome measures (OS: HR=2.55 [95% CI 1.36–4.78], PFS: HR=2.07 [95% CI 1.26–3.39]). Additionally, M-MDSCs showed strong predictive value for PFS across different cutoff determination methods (Cutoff method median: HR=1.96 [95% CI 1.40–2.72]; pooled different cutoff methods: HR=2.06 [95% CI 1.22–3.49]). Conversely, PMN-MDSCs were significant prognostic markers only in subgroups utilizing the median as the cutoff method, underscoring their potential as predictive markers in specific contexts (OS: HR=1.74 [95% CI 1.11–2.75]; PFS: HR= 2.29 [95% CI 1.39–3.77]).

### 3.5 Risk of bias

**Figure 4:**
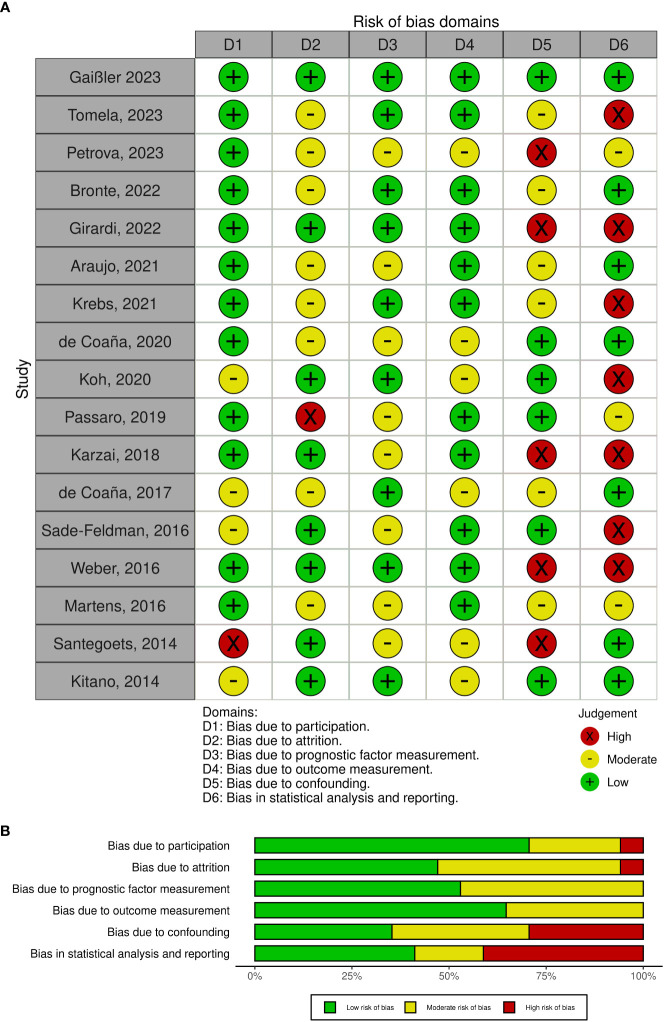
**(A)** Risk of bias summary plot. **(B)** Risk of bias assessment using the QUIPS tool.

Our assessment of the risk of bias across various categories yielded heterogeneous outcomes, as detailed in **Figures 4A, B**. A notable observation was that the employment of non-standardized markers significantly elevated the risk of bias within the prognostic marker category. Specifically, the study attrition and confounder categories were identified as areas with a particularly high risk of bias. This heightened risk was primarily attributed to inadequate descriptions of potential confounding variables or insufficient information regarding participants not included in the analysis. In the category of statistical analysis and reporting, a high risk of bias was frequently encountered; 8 out of 17 studies did not directly report hazard ratios, necessitating their estimation through the method proposed by Tierney et al. (25) or extracted from alternative sources. Specifically, hazard ratios were estimated for 6 studies using the Tierney et al. methodology, while for one study, hazard ratios were obtained from other published sources. Additionally, one study provided hazard ratios upon our direct request, highlighting the challenges and variability in data reporting practices across studies included in our meta-analysis.

### 3.6 Sensitivity analysis

To validate the reliability of our findings, we conducted a sensitivity analysis by excluding studies identified with a high risk of bias in any category. This stringent approach aimed to mitigate potential biases impacting our conclusions. Despite these exclusions, the pooled hazard ratios for both PFS and OS remained statistically significant, with PFS showing a hazard ratio of 2.59 [95% CI 1.73–3.87] and OS demonstrating a hazard ratio of 1.93 [95% CI 1.30–2.87]. However, heterogeneity remained in OS across all groups. This analysis was limited by the fact that PMN-MDSC or total MDSC could not be considered for PFS due to the lack of studies with low risk of bias here. The sensitivity analysis continues to show significant results, especially for the M-MDSC in OS: 2.23 [95% CI 1.49–3.35]. However, we observed persistent heterogeneity in OS across all evaluated groups, indicating variability that could not be fully accounted for by excluding studies with high bias risk.

A limitation of our sensitivity analysis emerged when considering the specific subtypes of MDSCs, particularly PMN-MDSC and total MDSC, for PFS outcomes. The absence of studies with a low risk of bias for these subgroups precluded their evaluation, underscoring a gap in the available literature. Despite these constraints, the sensitivity analysis underscored the significance of M-MDSC in predicting OS, with a hazard ratio of 2.23 [95% CI 1.49–3.35], reinforcing the potential prognostic value of this MDSC subtype in the context of immune checkpoint inhibitor therapy.

### 3.7 Publication bias

**Figure 5:**
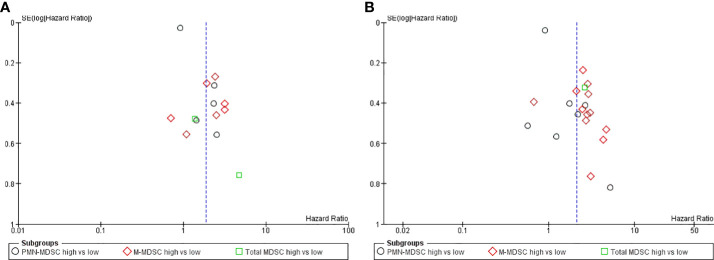
**(A)** Funnel plot progression free survival: Assessing the publication bias, the hazard ratio was plotted on the X-axis and the standard error corresponding to the logarithm of hazard ratio on the Y-axis. **(B)** Funnel plot overall survival: Assessing the publication bias, the hazard ratio was plotted on the X-axis and the standard error corresponding to the logarithm of hazard ratio on the Y-axis.

The analysis of OS and PFS data revealed asymmetry in the funnel plots, indicative of potential publication bias or other small-study effects (**Figures 5A, B**). This observation was further substantiated by the results of the Egger test, which demonstrated statistical significance with P<0.001 for PFS and P< 0.001 for OS, suggesting the presence of bias in the reported. In alignment with our predefined protocol, we employed the trim-and-fill method as a corrective measure to address this asymmetry, aiming to estimate the effect of potentially unpublished studies on our meta-analysis outcomes. The application of this method led to adjusted hazard ratios (HR) of 1.72 (95%CI 1.23, 2.41) for the PFS and 1.89 (95%CI 1.39, 2.58) for the OS. These revised estimates further underscore the robustness and statistical significance of our findings, reinforcing the predictive value of MDSC levels on the outcomes of patients undergoing immune checkpoint inhibitor therapy, even after accounting for potential publication bias.

## 4 Discussion

In our systematic review, we included 17 studies from which we were able to extract data for a meta-analysis. We included data from patients with melanoma (29–31, 34, 36, 37, 41–43, 45), NSCLC (33, 38, 39) prostate cancer (40, 44) and urothelial carcinoma (32). One study pooled data from solid tumors encompassing patients with 11 tumor entities (35).

Our findings indicate that MDSCs can predict both survival and response to ICIs, according to our endpoints of OS and PFS. Particularly, M-MDSCs are shown to be both a predictive and a prognostic marker. Analysis of the sub-cell populations revealed that M-MDSCs are significantly inversely correlated with OS and PFS. Although PMN-MDSCs did not reach statistical significance in our analysis, a trend was observed for both OS and PFS, suggesting the entire MDSC population as significant.

This meta-analysis aligns with previous meta-analyses that investigated MDSCs as predictive and prognostic markers in patients predominantly undergoing chemotherapy (9, 46).

The study of Koh et al. highlighted that M-MDSCs have even better predictive power of response and survival in NSCLC patients than PD-L1 expression on tumor cells (39), suggesting that MDSCs could enhance the predictive accuracy for OS and response rate across various tumor entities. Thus, the currently established markers such as PDL (in NSCLC) or LDH (in melanoma) could be supported by MDSC to predict a better correlation for OS and response rate in other tumor entities, as it has already been shown that patients (NSCLC) with a low PDL status also benefit from ICI (47).

The significance of our findings is underscored by Krebs et al. (34), who identified a subgroup of clinical non-responders to ICI, with an immune profile akin to responders, thus exhibiting prolonged OS. Similarly, Tomela et al. (29), found an inverse correlation between PMN-MDSC levels and PFS in the responder group.

Further research with a standardized gating strategy is crucial for establishing validated cut-off limits for MDSCs, thus facilitating their application in clinical practice as a dynamic prognostic marker. This could help identify patients who might benefit from ICI therapy or those at high risk of non-response, potentially making MDSCs a therapeutic target. The feasibility of this approach has been demonstrated by Tobin et al. (48). and the consideration of a cut-off value defined by healthy subjects as practiced by Kitano et al. (45) is also suggested.

Our analysis encountered significant heterogeneity, particularly notable within the PMN-MDSC subgroup. This variability arose partly from the diverse methods used across studies to define cut-off values, typically applying these thresholds as percentages of living peripheral blood mononuclear cells (PBMCs). Such methodological disparities were a key factor contributing to the observed heterogeneity.

Moreover, the landscape of immunotherapy, particularly with the introduction of ipilimumab in 2011, has undergone significant evolution. This breakthrough marked the beginning of an era characterized by the development and approval of various immune checkpoint inhibitors (ICIs), each with distinct indications. The evolving use of ICIs, including their combination therapies and earlier application in treatment protocols, has led to diverse study designs and populations. These changes reflect the dynamic nature of cancer treatment protocols and have inevitably influenced the heterogeneity observed in our meta-analysis.

This heterogeneity underscores the complexity of drawing generalized conclusions from the available data and highlights the need for standardized approaches in future research. This heterogeneity was addressed using a random effects model, as noted in the protocol, aiming to minimize its impact.

Lower MDSC correlated with a better prognosis, but it has not yet been possible to find a uniform cut-off value to use and implement the cell population as a clinically marker.

Subgroup analyses revealed significant effects for PFS and OS for both M-MDSC and PMN-MDSC populations when studies utilized consistent methods for determining cutoffs, with low heterogeneity. Notably, heterogeneity was attributed not only to tumor entities but also to inherent characteristics within each entity, though limited to NSCLC and melanoma due to the number of studies. Variations in the methodologies used to categorize PMN-MDSC, particularly in terms of gating strategies and the markers employed, have contributed to the observed heterogeneity in our analysis. An illustrative example is the approach taken by Passaro et al. (38), which stood out by utilizing absolute cell counts instead of percentage values for defining PMN-MDSC levels. This deviation underscores the broader issue of inconsistency in measurement techniques across studies, which adds to the challenge of synthesizing data and drawing uniform conclusions.

Despite the significant heterogeneity introduced by such methodological differences, our rigorous assessment process confirmed that the study by Passaro et al. satisfied all predefined inclusion criteria. Therefore, in adherence to our commitment to a comprehensive and inclusive review, we retained the study by Passaro et al. within our meta-analysis. This decision reflects our endeavor to capture a wide spectrum of data and insights, even when faced with high heterogeneity, to ensure the robustness and breadth of our analysis.

In recent years, there has been a growing consensus regarding the identification of similar subpopulations of MDSCs using specific markers (10). This emerging agreement highlights the need for standardized markers to distinguish MDSC subpopulations clearly, especially to avoid confusion with neutrophils. A uniform marker, such as Lox-1, could serve this purpose effectively by providing a clear distinction. Additionally, the adoption of a myeloid score, which incorporates multiple validated markers as proposed by Huber et al., could offer a more nuanced understanding of MDSCs’ role within the immune system (49). Furthermore, considering the complex interplay within the immune system, an alternative approach involves using an index that not only assesses the immunosuppressive impact of myeloid cells but also includes cytotoxic cells. This comprehensive index, as utilized by Araujo et al. (35), offers a more holistic view of the immune landscape. Such an index has the potential not only to enhance our understanding of the immune system’s dynamics but also to serve as a valuable prognostic tool throughout the course of a disease. This multi-faceted approach acknowledges the intricate nature of immune responses and the importance of a comprehensive evaluation for both research and clinical applications (35). Here, the PMN-MDSC group is not significant, so they are not as immunosuppressive as initially expected and possibly the M-MDSC population takes on this characteristic. The two subgroups of MDSCs differ not only in terms of their phenotype, but also in terms of their mechanism of action: while PMN-MDSC are mainly antigen-specific, M-MDSC can be both antigen-specific and antigen-nonspecific (11). These differences may be the reason why PMN-MDSCs did not reach significance in this analysis: some studies have shown a stronger immunosuppressive capacity of M-MDSCs compared to PMN-MDSCs on T cells (16, 50, 51). This could explain the difference in significance in general, as T-cells are the main effectors in ICI. Bronte et al. (46) also note that M-MDSCs have been shown to have a continuous immunosuppressive effect on neoantigen-specific T cells (46, 52). Neoantigen load was negatively correlated with outcome in NSCLC patients (46, 53), suggesting that neoantigen inhibition is more relevant specifically in this tumor subgroup, which can only be addressed by M-MDSCs. This could be the reason for the altered behavior of PMN-MDSCs especially in this patient population. In addition, it should also be noted that the number of studies on PMN-MDSCs in this meta-analysis was relatively small and that the elimination of the study by Passaro et al. (38) as part of a sensitivity analysis increased the prognostic and predictive relevance of PMN-MDSCs.

Studies like Gaißler et al. highlight the prognostic significance of M-MDSC dynamics, showing that patients with initially high MDSC levels but subsequent reductions can achieve similar OS to those with consistently low levels (31). This is corroborated by findings from de Coaña (2017) et al.; after three weeks of therapy patients with lower M-MDSC had a better OS (HR= 2.89 (1.59–6.99) P= 0.002), nevertheless the baseline was not significant (36). The study conducted by Tarhini et al. (54) presents findings that align with a key observation: a significant reduction in the levels of total MDSCs is associated with improved PFS. This indicates that patients who experience a larger decrease in MDSC levels tend to have a longer period without disease progression, underscoring the potential role of MDSCs as dynamic biomarkers for treatment outcomes in cancer therapy.

The limitations of our study include several critical aspects that affect the interpretation and reliability of our results. Firstly, the variability of markers in our meta-analysis posed a significant challenge. Since 2016, there has been a convergence towards the use of standardized markers and gating strategies for MDSCs and their subpopulations, as recommended by Bronte et al. (10). Future research should adhere to these standardized markers to reduce variability. Secondly, our meta-analysis showed a high degree of heterogeneity, possible due to the use of different markers, study designs, and populations. We used a random effects model to address this issue. Furthermore, the assessment of risk of bias added complexity. The observed heterogeneous results necessitated a sensitivity analysis to assess the robustness of our results, particularly with regard to discrepancies in hazard ratio reporting. Seven of the 17 studies did not report hazard ratios with 95% confidence intervals. For one study we were able to obtain this by contacting the author, whereas for the remaining six studies we had to estimate it from Kaplan-Meier curves or other sources. This led to some uncertainty, which we considered in our risk of bias analysis. In addition, our analysis raised concerns about publication bias. Visual inspection of the funnel plot for OS and PFS indicated possible publication bias, which was confirmed by the Egger test. We estimated the impact of unpublished studies using Duval and Tweedie’s trim-and-fill method.

In conclusion, the role of MDSCs, especially M-MDSCs, has been increasingly recognized and validated in the context of cancer immunotherapy. These cells have emerged as significant prognostic markers for predicting the response to immune checkpoint inhibitors. Their utility extends beyond mere prognostication; MDSCs offer a window into identifying patients who may not initially respond to therapy based on their baseline myeloid cell profiles. Such insights are invaluable for tailoring treatment approaches, potentially guiding the escalation of therapy to overcome resistance mechanisms.

Furthermore, MDSCs present a dynamic aspect of the tumor microenvironment that could be monitored over the course of treatment. By observing changes in MDSC levels, clinicians can gain insights into treatment efficacy in real-time, allowing for adjustments to therapy that could enhance outcomes. The potential of MDSCs extends to their viability as therapeutic targets themselves, suggesting that manipulating their levels or function could directly improve the efficacy of ICIs.

Despite the promising horizon that MDSCs represent in the realm of cancer therapy, several challenges remain. A critical barrier to the clinical integration of MDSCs as a biomarker is the lack of standardization in identifying and quantifying these cells. The field would greatly benefit from consensus on the markers used to define MDSC subpopulations and uniform cutoff methods to categorize their levels accurately. Addressing these challenges through future research is essential to harnessing the full potential of MDSCs in improving patient outcomes. By establishing standardized methodologies and integrating MDSC assessments into clinical practice, we can move closer to a future where cancer therapy is more personalized, predictive, and potent.

## Data availability statement

The original contributions presented in the study are included in the article/**Supplementary Material**. Further inquiries can be directed to the corresponding author.

## Author contributions

MM: Writing – original draft. VO: Writing – original draft. VU: Writing – review & editing. SH: Writing – review & editing. VB: Writing – review & editing. CR: Writing – review & editing. JH: Writing – original draft. SS: Writing – original draft.
